# Mentorship and Ethics in Global Health: Fostering Scientific Integrity and Responsible Conduct of Research

**DOI:** 10.4269/ajtmh.18-0562

**Published:** 2018-11-14

**Authors:** Elizabeth A. Bukusi, Yukari C. Manabe, Joseph R. Zunt

**Affiliations:** 1 Center for Microbiology Research, Kenya Medical Research Institute, Nairobi, Kenya;; 2 Department of Global Health, University of Washington, Seattle, Washington;; 3 Department of Obstetrics and Gynecology, University of Washington, Seattle, Washington;; 4 Division of Infectious Diseases, Department of Medicine, School of Medicine, Johns Hopkins University, Baltimore, Maryland;; 5 Infectious Diseases Institute, Makerere University College of Health Sciences, Kampala, Uganda;; 6 Department of Neurology, University of Washington, Seattle, Washington;; 7 Department of Epidemiology, University of Washington, Seattle, Washington;; 8 Department of Medicine (Infectious Diseases), University of Washington, Seattle, Washington

## Abstract

Addressing ethical issues through mentorship is key to encouraging scientific integrity and increasing research capacity. Across the global health arena, mentorship requires helping mentees understand and negotiate the regulatory aspects of research—which can substantially differ even between countries with similar resources. Mentorship support spans across the research framework from obtaining ethical approval and ensuring scientific integrity, to determining authorship and disseminating study results—providing multiple opportunities to model ethical behavior for mentees. The power imbalances between the global north and south in accessing funding resources produce further challenges in setting the research agenda and for ensuring equity in the dissemination of research findings. Gender further complicates the aspiration for equity; the proportion of women in high administrative or research positions remains low. This study explores four specific mentoring case scenarios commonly encountered in the global health research field in low- and middle-income institutions.

## Introduction

The increasing globalization of commerce, education, and research has resulted in increasing collaboration across countries. One manifestation of increased collaboration in academia is the rise in number of scientific publications. Between 2003 and 2013, the number of scientific manuscripts published in any of the more than 17,000 peer-reviewed scientific journals monitored by SCOPUS increased from 1.1 million to nearly 2.2 million.^1^ Over this same timeframe, scientists from low- and middle-income country (LMIC) institutions increased their percentage of scientific and technical publications from 9.5% to 13.7%.^1^ In addition, between 1988 and 2013, coauthorship by authors from more than one country increased from 8% to 19% and U.S. and Chinese scientists have reached approximate parity in total number of publications, with each country contributing 18.8% and 18.2% of the world’s total science and engineering publications in 2013, respectively. With this increase in publications, has come increasing reports of scientific misconduct, as well as attention to how institutions and mentors can monitor for scientific misconduct and provide role modeling and training in the responsible conduct of research (RCR) to reduce misconduct in trainees, especially in countries that have limited infrastructure to detect, investigate, or penalize scientific misconduct.^2^

Providing research trainees with a framework of ethical standards for conducting research, especially in the global multidisciplinary and multinational arenas, is essential to ensure that research findings are reliable, that future scientists conduct science in an ethical manner, and that research advances our understanding of the world and its inhabitants while respecting and protecting human and animal subjects used in research. Responsible conduct of research is based on ethical behavior of scientists toward their research subjects and colleagues. Scientific integrity and institutionalization of research oversight includes monitoring, and training. The National Academy of Sciences, Engineering and Medicine, in their Consensus Study Report on fostering integrity in research, noted: “Practicing integrity in research means planning, proposing, performing, reporting, and reviewing research in accordance with the values described above [objectivity, honesty, openness, accountability, fairness and stewardship].”^3^ Perhaps equally important to practicing integrity, is providing a structure through which scientific misconduct can be identified, reported and addressed. The InterAcademy Council, a multinational organization of science academies, stated “while procedures and institutions [are necessary] to effectively investigate allegations of irresponsible research conduct and act on the results, efforts aimed at preventing irresponsible conduct and ensuring good practices through mentoring and education are ultimately more important.”^4^

## Historical perspectives

The World Medical Association developed the Declaration of Helsinki adopted by its assembly in Helsinki in 1964.^5^ This guide was specific for physicians regarding engagement of their patients in research. Subsequently, in 1982 the Council for International organizations in Medicine, using the Declaration of Helsinki as a reference, provided guidelines for the conduct of biomedical research involving humans conducted in LMIC settings, where greater disparities in health care are encountered than in more developed countries.^6^ Before the 1980s, few institutions in the United States had adopted institutional review boards (IRBs) to oversee research involving humans or animals to respond to concerns of scientific misconduct and had instead relied on independent monitoring and regulation of scientific activities. In the 1980s, the emergence of several cases of scientific misconduct in the press led to U.S. congressional hearings that resulted in the creation of federal and institutional standards to reduce scientific misconduct.^7^ In 1989, to ensure “that attention be directed toward scientific integrity in the conduct of research,” the U.S. National Institutes of Health (NIH) revised National Research Service Award institutional training grants “to require that a program in the principles of scientific integrity be an integral part of the proposed research training effort.”^8^ Since this revision, guidelines have been updated but some experts suggest that despite these guidelines, transgressions in scientific integrity continue to occur and consensus is lacking regarding how to teach RCR or measure the effectiveness of such teaching.^9^

## Globalization of Research

The globalization of research and publishing has also resulted in increased awareness of the disparity of RCR training opportunities in many countries. With the advent of AIDS, the Fogarty International Center increased research training support for international scientists to build capacity to respond to epidemics through new programs, such as the AIDS International Research Training Program, first offered in 1988, and the international Research Ethics Education and Curriculum Development award, first offered in 2000. With the implementation of these international training programs came increased attention to strengthening didactic and interactive RCR education for trainees conducting research in different international settings. Although many of the early diaspora of Fogarty research training programs have risen to leadership positions and have facilitated increased availability of RCR training at their universities and institutions, successive generations of trainees continue to struggle with many of the same issues touching multiple facets of research—from design to implementation, analysis, and dissemination of results.

One recent study of researchers in LMICs reported that a common perceived factor associated with high prevalence of research misconduct was the lack of institutional structures or systems to support and promote research integrity—such as offices to promote research integrity, develop and disseminate policies on research misconduct, and provide channels for whistleblowing when misconduct was detected.^10^ Many universities and training institutions in resource-rich countries have evolved over centuries and have thus had opportunities to refine teaching methodologies to maximize the benefits of teaching for different stages of the educational cycle. Through academic partnerships, which have typically been closely aligned with research training grants, some of these advances have trickled down to institutions in LMICs, where the time since the countries achieved independence has been shorter and as a result the opportunities in many countries to refine teaching methodologies are less plentiful. In addition, these institutions have limited resources and may not prioritize the development of such offices and systems.^11,12^

Regardless of the maturity of teaching, training, and research regulation programs, recent evaluations suggest that education in the RCR for science and engineering students, especially for undergraduates, remains inadequate—and poorer performance by international trainees on measures of baseline RCR knowledge suggest these trainees are even less exposed to RCR training in their home countries.^13^ To further complicate this issue, codes of responsible conduct differ across countries, although efforts in the past decade have resulted in increased international consensus on the basic principles of RCR.^4^

Realizing that addressing all aspects of RCR is beyond the scope of this manuscript, we chose four common areas for misconduct noted in the international literature^14,15^ that each of us has encountered while mentoring trainees in our programs: 1) plagiarism, 2) determining authorship, 3) the appropriate use of an IRB, and 4) imbalances of power, especially across genders. These four common concerns are encountered frequently when mentoring undergraduate, graduate, or postdoctoral trainees and represent areas where good mentorship can decrease scientific misconduct in new trainees and encourage the next generation of trainees to become leaders. We provide case studies to illustrate each topic area and discuss how mentoring and institutional structures can be harnessed to detect and address transgressions and, when instituted early enough, prevent scientific misconduct from arising.

The literature review was guided by the Delphi method. Through a series of phone and in-person conversations with the authors involved in all chapters of this manual, we discussed potential content of each chapter. For our chapter, we initially defined the four most common or challenging scenarios associated with mentor–mentee relationships across our international settings. Through identification of key articles by the panel of authors, PubMed search of key terms associated with each scenario, and review of relevant articles listed in the references of these articles, we arrived at a list of relevant articles for our chapter; these articles were then vetted by the panel of authors and relevant observations were included in this chapter.

## Plagiarism

### Case study 1.

The student had published one paper and needed a second to graduate. This was a requirement of the university adopted by the accreditation body to encourage publication and research output. After copying and pasting whole sections of the first paper into the second, he was surprised when he was notified after submission that this was self-plagiarism and was not acceptable. Shocked, the student wondered how he could be accused of plagiarism because he had written the first paper.

One of the most common issues affecting the integrity of science is plagiarism. The interpretation of plagiarism is nuanced across cultures, and current definitions include not only the copying of another person’s work without citation, thus implying it is one’s own work, but also “self-plagiarism,” or repeating portions of one’s prior work (some exception is made for repeating sections of previously published materials and methodology sections).^16^ In countries where English is typically a second language, many graduate students are required to write in English for purposes of thesis or manuscript publication; in this context, copying text is sometimes considered a compliment to the original author of the plagiarized text.^17^ In addition, the type of publication may influence the likelihood of plagiarism; one study in India noted that plagiarism was highest in review articles and was not detected in any of the case reports reviewed.^18^

One study that interviewed Chinese supervisors about reasons for students copying text and how to remedy this issue concluded there were four major reasons for text-based plagiarism: 1) insufficient understanding of academic writing, 2) difficulty with the English language, 3) “shortage in intellectual and cognitive depth needed for handling a subject matter,” and 4) lack of training in the ethical conduct of research.^19^ Reasons given by students for plagiarizing included: the “normalcy of plagiarism” in their home environment; vague or nonexistent policies regarding RCR and plagiarism; and, for non-native English speakers, difficulty expressing oneself in English.^20^

The emergence of online programs to check for plagiarism, such as iThenticate, TurnitIn, and CrossCheck, have resulted in increased ease of assessing copied text; it is used by teachers and students from secondary school through university and professional levels. After the initial use of CrossRef plagiarism screening service, one prestigious scientific journal in China detected 692 (31%) of 2,233 submissions with “unoriginal material” and noted that “in ancient China…students were typically encouraged to copy the words of their masters.”^21^ However, these programs are not free and can have high levels of “false positives”—they will detect similarities in text in any section of a manuscript, including the reference section. One study that examined the frequency of detection of similar text in two entire issues of their journal found that sensitivity for fraud detection was improved, and false positive reports were decreased by adjusting the software to exclude review of bibliography, materials and methods sections, and reporting only similarity of sources greater than 2%.^22^

To successfully reduce the incidence of plagiarism among postgraduates, one group developed a module that included a didactic presentation about plagiarism followed by discussion of an anonymous paper that contained plagiarism detected by TurnItIn software. In this scenario, students were asked to discuss if plagiarism had occurred and what action should be taken against the student at that time or if similar reports of plagiarism occurred in the future with the same student.^23^ In a review of randomized interventions that attempted to improve RCR, the likelihood of committing plagiarism was reduced through practical exercises and integration of software to detect text matching.^24^ Other effective methods for decreasing the occurrence of plagiarism include scientific writing courses, modules on plagiarism in RCR courses, incorporating discussion of scientific integrity into the culture of working groups, and adhering to internationally recognized guidelines for scientific writing. Gasparyan and others^25^ provided a thoughtful review of plagiarism in the scientific setting and note that adherence to guidelines for authors and editors published by the Committee on Publication Ethics and the International Committee of Medical Journal Editors (ICMJE) could lead to marked reductions in plagiarism. One laboratory director noted that he posted a page containing an “ethical code of research conduct for university academics” in his laboratory, thus establishing an expectation for scientific behavior in his laboratory.^19^ Mentors can encourage responsible writing through teaching as they edit by providing trainees with written and oral feedback on written materials during one-on-one sessions, and explicitly discussing appropriate citation of others’ work during group meetings. With consideration for the yearly turnover of trainees, these types of discussions must be repeated with each new cohort.

## Authorship

### Case study 2.

Nearing the completion of a thesis, a postgraduate student received upsetting news. The student’s work had led to excellent findings that would add to the field of knowledge, but the supervising professor had recently informed the student that the work was to be published with the supervisor as first author. The supervisor needed first author papers for promotion. The student, they argued, only needed to publish for purposes of graduating and be done with the work. The supervisor rationalized that the student intended to focus on clinical work anyway and was not on an academic track. Regardless, the postgraduate scholar had put in the most work, from conceptualizing the research idea, to collecting and analyzing data, and drafting the manuscript. True, the guidance provided by the supervisor was key to completion of his training and yes, even the publication of the paper. But was it enough for the supervisor to take away the first authorship?

The ICMJE has created a widely accepted definition of authorship that is “intended to ensure that contributors who have made substantive intellectual contributions to a paper are given credit as authors, but also that contributors credited as authors understand their roles in taking responsibility and being accountable for what is published.”^26^ They propose that authorship be based on four criteria:

1. *Substantial contributions to the conception or design of the work; or the acquisition, analysis, or interpretation of data for the work*
2. *Drafting the work or revising it critically for important intellectual content*
3. *Final approval of the version to be published*
4. *Agreement to be accountable for all aspects of the work in ensuring that questions related to the accuracy or integrity of any part of the work are appropriately investigated and resolved.*

In a questionnaire study of 607 corresponding authors from LMICs, 77% reported that guest authorship, or adding an author who had not substantially contributed to the manuscript, took place at their institution, despite awareness of ICMJE recommendations regarding the responsibilities of authorship.^10^ This same publication noted that ghost authorship, or omitting an author who had contributed significantly, such as a professional writer, although less common, occurred at slightly more than 40% of their institutions.

The challenge with authorship of publications then is not the lack of clarity on who or what participation should qualify one to be an author, but the implementation of existing authorship guidelines. Fairness and integrity when attributing authorship should be guiding principles, especially when considering the unequal relationships of mentor and mentee and the lack of role models who not only talk the talk but walk the walk. Mentorship that upholds and encourages appropriate attribution of authorship can produce a critical mass of the next generation of scientists who will continue to uphold these values, as well as disseminate these same values into environments where inappropriate attribution still exists. One method to increase the likelihood of appropriate attribution of authorship is to ask mentors to provide guidance on authorship, from the initial design stage through the analysis and writing phases; discussions should include internationally recognized criteria required for authorship, author order, as well as guest and ghost authorship. In addition, traditional RCR courses often include sections on authorship.

Conflicts in authorship are best avoided through early discussions and authorship contracts—which should be based on internationally recognized guidelines and include author order. When authorship conflicts do arise, they are often affected by perceived repercussions that might occur—especially if the junior scientist is raising the concern. Resolution of conflicts can occur through direct dialogue between authors, mediation via an ombudsman office (if present at the university), or peer panels.

## Appropriate use of IRBs

### Case study 3.

A graduate student sought consultation on a matter concerning research sites. The scholar had been given approval from an accredited ethics review committee (ERC) to conduct research in a certain facility. However, at the time of data collection, they realized that the facility no longer had the patient flow or number of specimens needed for the study. On consultation with the graduate supervisor, the supervisor told the student that it was fine to go ahead and collect the data from a different location and when the thesis was written up, an explanation could be provided regarding reasons for the change in location. The supervisor noted there was no potential harm to any participants because the experiments were carried out on left over specimens after the tests needed for clinical care had been completed.

Research integrity laws that make research illegal without the approval of an IRB have been lagging in many LMICs. Although there has been a strengthening of research regulatory systems, not all LMICs have robust systems for continuing education of researchers, clear mechanisms or guidance for review of research protocols, or mechanisms for monitoring the implementation of research with corresponding procedures for dealing with violations of ethical guidelines for human subject protection.

There is general global consensus on the need to obtain ethical clearance from an IRB or research ERC for investigators developing and implementing a research protocol, especially when it involves human or animal subjects.^6^ There has been clear guidance from the U.S. Public Health Service regarding expectations of how research is defined and what expectations are required to receive research funding from the U.S. government, but these may not be applicable to funding awarded through other sources.^27^

Although research is highly valued for innovation and finding new solutions to challenges, an additional benefit are the Facilities and Administrative, or “indirect” costs that are provided with nearly all research grant awards to institutions in the United States and Europe. These indirect funds provide institutions with financial resources to administer grants and strengthen institutional programs to support research, such as IRBs and integrated programs in grant writing and RCR training. As grants directly awarded to institutions in LMICs are relatively new, similar indirect costs have unfortunately not been available to support the development of similar research infrastructure or the human resources required for research regulation and administration.

Training and mentoring on conduct during the implementation of research therefore remains important. Examples of potential breaches of IRB/ERC approval include but are not limited to: change in an approved location of study implementation, change in the type of sample needed for the research procedures, change in wording used for the consenting of participants for prospective studies, or the amount of compensation provided for participation in research. The standards of expectations on the appropriate use and communication with the IRB/ERC continue to evolve. When mentors are not well versed with the current local and relevant international regulations governing biomedical research, mentees can find themselves in the crosshairs between the mentor and the IRB/ERC.

## Imbalances of power

### Case study 4.

A junior female scientist who was a regional expert in a particular area, was asked to be the in-country principal investigator (PI) for a research study for which a grant had already been written and funded by an international funding agency. The in-country PI worked hard to obtain the necessary bioethical approvals, enlist staff, conduct study training, and ultimately recruit participants and collect study data. Ultimately, the final paper was written and the in-country PI was included as a middle author, after never being asked to comment on the content of the paper other than to provide final approval.

Here, the in-country PI was being treated as solely an implementer and had no role in the design of the study nor of the original research question—which may or may not have had any significant local or regional relevance. Because the international PI had brought the funding, there was a clear imbalance of power that was antithetical to the “collaborative partnership.” Although the in-country PI was included as a coauthor, she did not have a scientific voice, was being held to a low scientific standard, and was recognized only for field implementation. The opportunity for a more equitable scientific role in this situation was absent largely due to failure to provide a venue for scientific exchange and collaboration. The tokenism of the authorship in this example is also subliminally condescending and displayed a lack of trust. Importantly, the in-country PI likely had to negotiate resource limitations, social, and political contextual complexities without which the project may never have been completed and are, therefore, equally important to the success of the project.^28^

This case study highlights the need for mentors from both the global north and global south to ensure that, “[a researcher’s primary ethical obligation in…global health experiences is to improve the health and well-being of the individuals and communities they visit.”^29,30^ Often as a result of funding imbalances, because researchers in the global south still do not have sufficient access or experience to acquire local or international research funding, mentors must include lessons on the importance of the integration of local relevance to balance decision making in a way that empowers local investigators.

An additional aspect of this case study that may have also played a role is gender. As the percentage of women who enter medicine and science increases, many seek to also enter academic research. In a survey of 1,719 new recipients of NIH career development (K) awards, 150 (30.4%) of the 493 female respondents reported having experienced sexual harassment including: sexist remarks or behavior, unwanted sexual advances, subtle bribery to engage in sexual behavior, threats to engage in sexual behavior, or coercive advances.^31^ To compound this issue, nearly all countries have gender inequity in leadership positions, and women in many countries still experience outright hostility, sexual harassment or marginalization in the offices, clinics, and hospital environments in which they strive to work. These issues tend to be magnified in LMIC research settings where the roles of women have not traditionally included the position of PI, dean, or rector. A growing number of activities to increase the visibility of women in scientific leadership positions, such as Women Leaders in Global Health, and the L’Oreal-UNESCO for Women in Science Awards, are increasing recognition of women’s leadership potential and providing opportunities for women to develop networks of female mentor leaders to assist the transition to more equitable leadership of research and scientific programs. Although the #MeToo movement has affected many areas of commerce and entertainment, attention to sexual harassment within science has lagged behind.^32^ Dr. Kathryn Clancy, author of this article, noted “many science workplaces use legal definitions of sexual harassment to set the standard for workplace conduct. If that is the bar that has to be met for a disgusting behavior to be considered actionable by a university, research institute, or field station, it is a high one. An enormous range of disrespectful and even frightening behavior can slip under that bar, even though it damages the careers of victims and bystanders, holding back scientific advancement.”^32^ The National Academies of Sciences, Engineering, and Medicine recently released a Consensus Study Report detailing the extent to which women in the fields of science, engineering, and medicine have been affected by sexual harassment and identifies methods to address harassment in these settings.^33^

To increase equity across collaborations, institutions must change existing research and training cultures to bolster the ability of collaborators to participate in all stages of research—from development of research ideas and protocols to implementation, analysis, and dissemination of study results. Increasing global awareness of gender inequity and sexual harassment should also lead to change—through pressure on institutions and individuals to enforce existing rules and regulations that are relevant to gender equity and anti-harassment or to implement new rules and regulations in places where none currently exist.

In conclusion, the need for research integrity as a core part of mentoring is an important undertaking that should be implemented from the conception of the research idea, through the development, implementation, and dissemination of study results. There is a great need for mentors to be aware of and deal with the challenges that mentees are likely to face throughout the research process. The Mentoring Competency manuscript in this supplement includes other core—and key—competencies that mentors should strive to achieve to ensure their trainees receive outstanding mentoring.^34^ Ethics in research is not just a question of following the letter of the law, but represents a way of conducting oneself with integrity and as a scientist who provides a model that sets an ethical course for others to follow. We need a critical mass of mentors who model ethical conduct along the entire cascade of research and who insist on the conduct of locally relevant research. The mentorships workshops were not designed to evaluate the before or after practice within the institutions where they were conducted. This is a limitation which will be addressed in future workshops where specific attention will be focused on pre- and posttest evaluations on specified domains of practice with regards to ethical conduct.

If education is a life-long process, then mentoring and ethics are key pillars of this journey, and both can be harnessed to increase scientific integrity and research that moves the needle of public health toward equality for all.
